# PM2.5 promotes human bronchial smooth muscle cell migration via the sonic hedgehog signaling pathway

**DOI:** 10.1186/s12931-017-0702-y

**Published:** 2018-03-02

**Authors:** Xiuqin Ye, Wei Hong, Binwei Hao, Gongyong Peng, Lingmei Huang, Zhuxiang Zhao, Yumin Zhou, Mengning Zheng, Chenglong Li, Chunxiao Liang, Erkang Yi, Jinding Pu, Bing Li, Pixin Ran

**Affiliations:** 1 State Key Laboratory of Respiratory Diseases, Guangzhou Institute of Respiratory Disease, The First Affiliated Hospital of Guangzhou Medical University, Guangzhou, China; 2 GMU-GIBH Joint School of Life Sciences, Guangzhou Medical University, Guangzhou, Guangdong China; 3 Xiamen Humanity Hospital, Xiamen, Fujian China; 4 Respiratory Department of the First Hospital of Yueyang City, Yueyang, Hunan China; 5 The First Affiliated Municipal Hospital, Guangzhou Medical University, Guangzhou, Guangdong China

**Keywords:** COPD, PM2.5, Human bronchial smooth muscle cell, Migration, Sonic hedgehog signaling pathway

## Abstract

**Background:**

The contribution of airway remodeling in chronic obstructive pulmonary disease (COPD) has been well documented, with airway smooth muscle cell proliferation and migration playing a role in the remodeling process. Here, we aimed to verify the effects of fine particulate matter (PM2.5) on human bronchial smooth muscle cell (HBSMC) migration and to explore the underlying signaling pathways.

**Methods:**

HBSMC apoptosis, proliferation and migration were measured using flow cytometry, cell counting and transwell migration assays, respectively. The role of the hedgehog pathway in cell migration was assessed by western blotting to measure the expression of Sonic hedgehog (Shh), Gli1 and Snail. Furthermore, siRNA was used to knock down Gli1 or Snail expression.

**Results:**

PM2.5 induced HBSMC apoptosis in a dose-dependent manner, although certain concentrations of PM2.5 did not induce HBSMC proliferation or apoptosis. Interestingly, cell migration was stimulated by PM2.5 doses far below those that induced apoptosis. Additional experiments revealed that these PM2.5 doses enhanced the expression of Shh, Gli1 and Snail in HBSMCs. Furthermore, PM2.5-induced cell migration and protein expression were enhanced by recombinant Shh and attenuated by cyclopamine. Similar results were obtained by knocking down Gli1 or Snail.

**Conclusions:**

These findings suggest that PM2.5, which may exert its effects through the Shh signaling pathway, is necessary for the migration of HBSMCs. These data define a novel role for PM2.5 in airway remodeling in COPD.

**Electronic supplementary material:**

The online version of this article (10.1186/s12931-017-0702-y) contains supplementary material, which is available to authorized users.

## Background

Chronic obstructive pulmonary disease (COPD) has become an increasingly serious threat to human health, but its pathogenesis remains poorly understood. Airway remodeling is widely recognized as the core hallmark of chronic bronchiolitis leading to COPD [1]. Many studies have shown that COPD is associated with airway wall thickening [2–4]. Moreover, Dournes and colleagues have shown that the progression of airflow obstruction in COPD is strongly associated with thickening of the smooth muscle layer [5]. The abnormal migration of airway smooth muscle cells (ASMCs) is thought to play a role in airway remodeling [6]. Biopsy sections from COPD and asthma patients have shown that the smooth muscle mass moves closer to the epithelium [7, 8]. The causes of these abnormalities are complex.

Air pollution is an increasingly serious problem, with environmental particulate matter (PM) posing a significant hazard to human health, especially in developing countries. Furthermore, our previous study established an association between exposure to ambient PM and COPD [9]. PM with an aerodynamic diameter smaller than 2.5 μm (PM2.5) can deposit in the respiratory tract and enter alveolar cells, inducing or worsening pulmonary diseases. Road traffic is known to be a major source of PM2.5 in urban environments, while residential biomass (e.g., wood, straw and animal feces) combustion for cooking or heating contributes considerably to indoor PM2.5 exposure in many rural environments. Investigating the effects of traffic ambient PM2.5 (TAPM2.5) and wood smoke PM2.5 (WSPM2.5) on human bronchial smooth muscle cells (HBSMCs) is therefore imperative for providing experimental evidence for the effects of different types of PM2.5 on airway remodeling.

Increasing evidence suggests that the Sonic hedgehog (Shh) pathway is involved in adult lung diseases such as pulmonary fibrosis, asthma, COPD and lung cancer [10]. The hedgehog (Hh) family of extracellular signaling proteins includes Shh, Indian hedgehog (Ihh) and Desert hedgehog (Dhh). Shh is the most broadly expressed and the most potent member of this family [11]. The presence of Shh releases Smoothened (SMO) from the inhibition exerted by the Patched (Ptch) receptor. SMO is a G protein associated with a receptor that transduces the Shh signal inside the cell. Activation of the Shh pathway results in nuclear translocation of the transcription factor Gli, which regulates the expression of genes involved in cell differentiation, survival and growth, such as cyclins and Snail, as well as Hh pathway genes including Gli and Ptch [12]. Three Gli genes (Gli1, Gli2 and Gli3) have been identified in mammalian tissues, of which Gli1 is considered the only specific marker of Shh pathway activity [13]. Several recent studies have shown that the Shh pathway is associated with cancer cell migration in pancreatic, ovarian and gastric carcinomas [14–16]. However, to date, the role of the Shh pathway in HBSMC migration has not been elucidated. Furthermore, the exact regulatory mechanisms by which the Shh pathway affects HBSMC migration remain unclear. A recent study has shown that Snail genes act primarily as survival factors and as inducers of cell migration, rather than as inducers of epithelial-mesenchymal transition (EMT) or cell fate [17]. Thus, further investigation of the mechanisms of HBSMC migration is required.

In this study, we tested the hypothesis that PM2.5 induces HBSMC proliferation, apoptosis and migration. We found that PM2.5 induces HBSMC migration and Shh pathway activation at concentrations far lower than those required for inducing apoptosis. We further confirmed that the Shh signaling pathway was required for the PM2.5-dependent migration of these cells.

## Methods

### Cell culture

HBSMCs and human bronchial fibroblasts (HBFs) were purchased from ScienCell (Carlsbad, CA, USA). These cell lines were cultured in the recommended medium supplemented with 2% fetal bovine serum and 1% growth supplement, and maintained at 37 °C in a humidified atmosphere with 5% CO_2_. The medium was replaced every 2 days. Cells from passages 3–8 were used for experiments.

### Particle collection and preparation

TAPM2.5 was collected from a busy road beside Dongfeng West Road, Guangdong City, Guangzhou Province (traffic jam hours, 8:00–21:00 during March–April 2015). Each filter membrane was collected after about 13 h and the machine was placed 3 m away from the road edge and 2 m away from the ground. WSPM2.5 was collected from the burning of China fir in a conventional Chinese wood stove (April 23–May 6, 2015), as previously described [18]. Each filter membrane was collected after about 1 h at full combustion and the machine was placed 2 m away from the oven. Both PMs were collected with a high-volume sampling machine (TE-6070, Tisch, USA) equipped with a PM2.5 selective-inlet head (1.13 m^3^/min). TAPM2.5 and WSPM2.5 were collected on glass fiber membrane filters with a 1.6-μm pore size and a 406-cm^2^ sampling area. The amount PM2.5 was defined as the weight increase of each filter. The filters were soaked in dimethyl sulfoxide (DMSO), and the solutions were then filtered through a 5-μm needle filter. The supernatant was collected, and the particles recovered from different filters were pooled to ensure a homogenous batch of particles. The 15 mg/ml TAPM2.5 and 40 mg/ml WSPM2.5 stock solutions were stored at − 20 °C until use. In the control group, filters without particles were prepared in the same conditions containing 0.1% DMSO. The final concentration of DMSO in the well did not exceed 0.5%. The presence of DMSO did not affect cell viability.

### Flow cytometry

HBSMCs were treated with TAPM2.5 (0, 15, 30, 60, 120, 240 and 480 μg/ml) or WSPM2.5 (0, 3, 6, 12, 24, 48 and 96 μg/ml) for 72 h. Cells were then harvested, washed and resuspended in phosphate-buffered saline (PBS). Apoptotic cells were identified using an annexin V-fluorescein isothiocyanate (FITC)/propidium iodide (PI) cell apoptosis kit (Invitrogen, Carlsbad, CA, USA) according to the manufacturer’s protocol. Briefly, the cells were washed and subsequently incubated with 100 μl of 1× annexin binding buffer containing 5 μl of annexin V-FITC and 1 μl of PI for 15 min in the dark. Apoptosis was then analyzed by flow cytometry (BD Biosciences).

### Cell proliferation assays

Cell proliferation was analyzed by cell counting and EdU immunostaining. For cell counting, 7.5 × 10^4^ HBSMCs were plated in 12-well plates in 1 ml of smooth muscle growth medium (SMGM). The following day, the cells were washed and transferred to 0.2% FBS-containing DMEM-F12 for 24 h, and subsequently treated with TAPM2.5 (15 μg/ml) or WSPM2.5 (3 μg/ml) for an additional 48 h. At the end of each experimental period, the cells were harvested with trypsin-EDTA and counted using a Zeiss Coulter Counter. At least three wells were analyzed per condition per experiment. For the analysis of cell proliferation by EdU immunostaining, a Cell-Light™ EdU staining kit was used to label the nuclei of dividing cells. HBSMCs were seeded in triplicate into 96-well plates at a density of 5000 cells per well and cultured in SMGM overnight. The cells were then treated with TAPM2.5 (15 μg/ml) or WSPM2.5 (3 μg/ml) for 24 h, and then subjected to EdU staining. EdU-incorporation assays were performed according to the manufacturer’s protocol [19, 20]. Briefly, 100 μL of 50 μM EdU were added to each well for 4 h. The cells were fixed in 4% paraformaldehyde and incubated with 2 mg/ml aminoacetic acid for 5 min with constant shaking. The cells were then incubated with 100 μL of penetrant per well for 10 min with constant shaking, followed by 100 μL of 1× EdU solution for 30 min. Hoechst 33,342 was used to stain cell nuclei. Images were acquired using a fluorescence microscope equipped with a × 20 objective. In each experiment, the proportion of EdU-positive cells was determined by analyzing cells from three random fields.

### Cell migration assays

Cell migration was assessed using the transwell chamber method. Assays were performed using Corning cell culture inserts (6.5 mm diameter, 8-μm pore size). TAPM2.5 (15 μg/ml) or WSPM2.5 (3 μg/ml) was prepared in DMEM-F12 supplemented with 10% FBS and added to the lower chamber. HBSMCs or HBFs (1 × 10^5^) in serum-free DMEM-F12 were added to the upper chamber. After a 6-h incubation at 37 °C, cells on both sides of the membrane were fixed and stained with 0.1% crystal violet solution. Cells on the upper side of the membrane were then removed with a cotton swab. Cell migration was evaluated by counting the number of cells that migrated to the lower side of the membrane in five random fields using a microscope equipped with a × 20 objective. In some experiments, 100 ng/ml recombinant Shh protein (r-Shh; R&D Systems, Minneapolis, MN, USA) was added to the lower chamber. In experiments where the SMO inhibitor, cyclopamine (Sigma-Aldrich, St. Louis, MO, USA), was used, cells were pretreated with the drug for 30 min prior to PM2.5 or r-Shh exposure.

### Western blotting

HBSMCs were harvested in RIPA cell lysis buffer supplemented with protease inhibitors (Thermo, Rockford, IL, USA), and protein concentrations were determined using the BCA protein assay. Protein extracts (40 μg) were separated by SDS-PAGE using 10% polyacrylamide gels, and then transferred to polyvinylidene difluoride (PVDF) membranes. Membranes were blocked with PBS/0.5% Tween 20 containing 5% skim milk, incubated overnight at 4 °C with primary antibodies against Shh, Gli1 and Snail (Abcam, Cambridge, MA, USA), and then incubated with a horseradish peroxidase-conjugated goat anti-rabbit IgG antibody (Proteintech) for 1 h at room temperature. Immunoreactivity was detected using an enhanced chemiluminescence kit according to the manufacturer’s instructions. Protein expression levels were normalized against GAPDH expression.

### RNA interference

Gli1, Snail and negative control (NC) siRNAs were purchased from Life Technologies. HBSMCs were grown in six-well plates to 60% confluence, and then transfected with 50 nM siRNA using 7.5 μL Lipofectamine™ RNAiMAX according to the manufacturer’s instructions. Gli1 or Snail knockdown efficiency was evaluated 72 h post-transfection by western blotting.

### Statistical analysis

Data are expressed as the mean ± SD of at least three independent experimental repeats. Comparisons between groups were performed using one-way ANOVA and Student’s *t*-test. A value of *P* < 0.05 was considered statistically significant.

## Results

### Effects of PM2.5 on the proliferation and apoptosis of HBSMCs

**Fig. 1:**
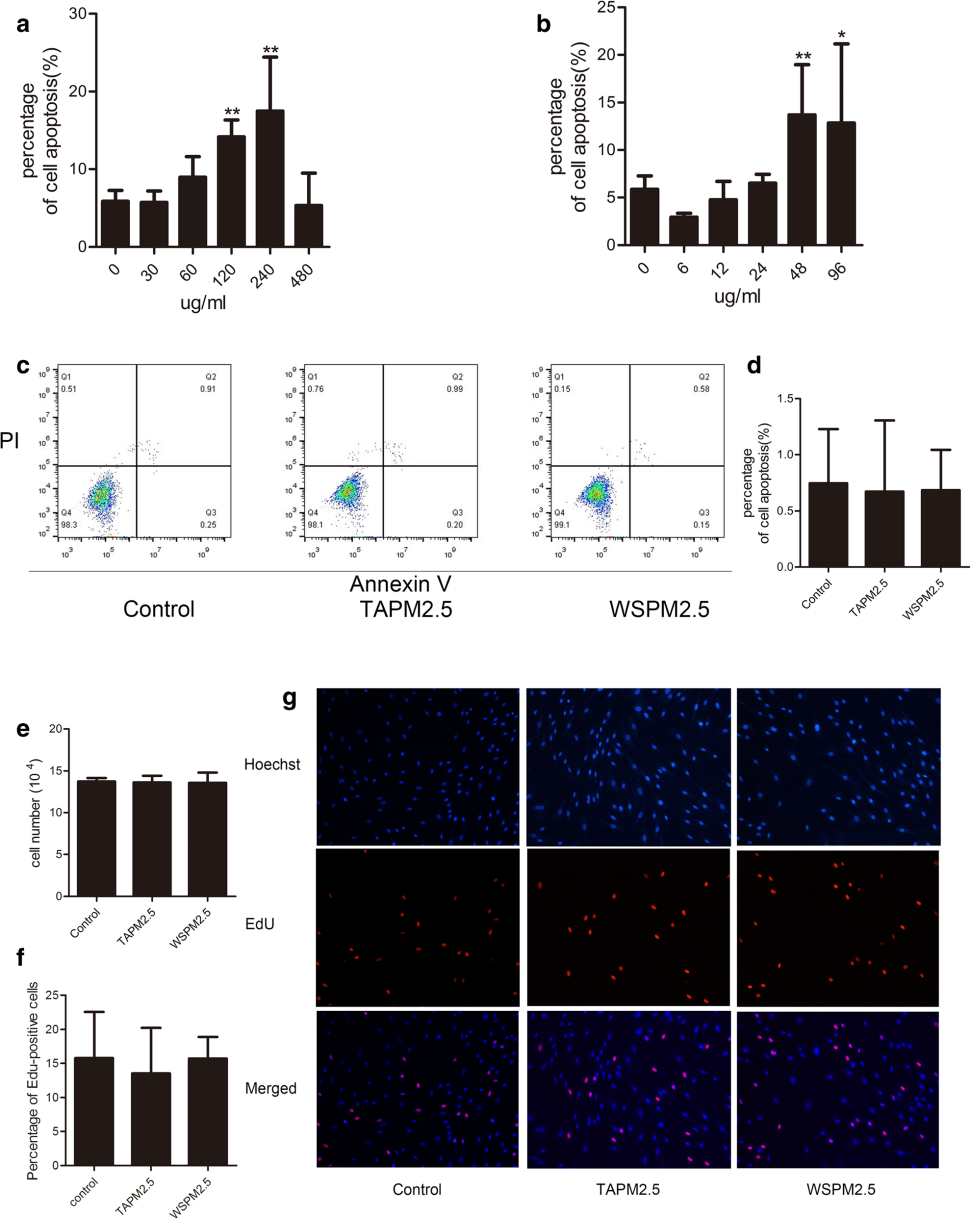
Effects of PM2.5 on HBSMCs. **a** Effects of various TAPM2.5 concentrations on HBSMC apoptosis. **b** Effects of various WSPM2.5 concentrations on HBSMC apoptosis. **c** and **d** Effects of PM2.5 (15 μg/ml TAPM2.5 or 3 μg/ml WSPM2.5) on HBSMC apoptosis. **e** Effects of PM2.5 on HBSMC number. **f** and **g** Evaluation of cell proliferation by EdU incorporation. *, *P* < 0.05; **, *P* < 0.001 compared with the control

Cell apoptosis assays were performed to evaluate the effects of PM2.5 on cell survival. HBSMCs were treated with varying concentrations of TAPM2.5 or WSPM 2.5 for 72 h before evaluation of apoptosis by flow cytometry. PM2.5 treatment increased apoptosis in HBSMCs (Fig. 1a and b). The proportion of apoptotic cells observed following treatment of HBSMCs with 120 μg/ml and 240 μg/ml TAPM2.5 was 29.24 ± 3.47% and 34.64 ± 7.51%, respectively. Following treatment with 48 μg/ml and 96 μg/ml WSPM2.5, the proportion of apoptotic cells was 23.57 ± 6.05% and 23.09 ± 12.37%, respectively. These values differed significantly from that of control HBSMCs, where 14.00 ± 2.92% of cells were apoptotic. However, treatment of HBSMCs with TAPM2.5 concentrations above 240 μg/ml resulted in a decrease in apoptosis to a level similar to that observed in the control population. Far lower doses of TAPM2.5 (15 μg/ml) or WSPM2.5 (3 μg/ml) had no significant effect on HBSMC apoptosis (Fig. 1c and d).

Because little is known about the effects of PM2.5 on the growth of HBSMCs, we next investigated the effects of PM2.5 on cell proliferation. As shown in Fig. 1e–g, exposure to 15 μg/ml TAPM2.5 or 3 μg/ml WSPM2.5 had no significant effect on the proliferation of HBSMCs. Taken together, our results clearly established that low concentrations of TAPM2.5 or WSPM2.5 do not induce HBSMC proliferation and apoptosis, but high PM2.5 concentrations induce apoptosis.

### Effects of PM2.5 on the migration of HBSMCs and HBFs

**Fig. 2:**
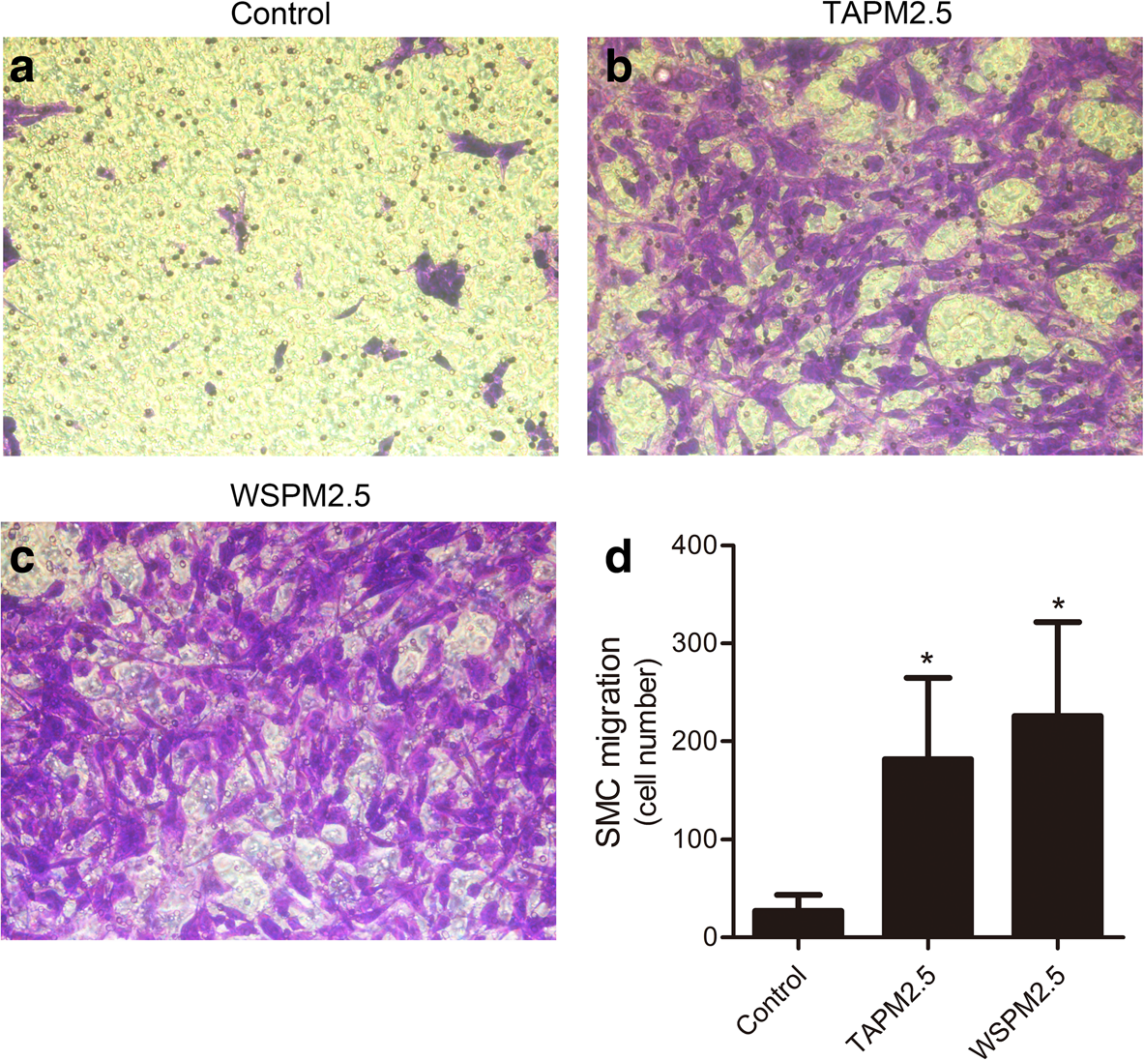
Effects of PM2.5 on HBSMC migration. **a** HBSMC migration in the control group. **b** Effects of TAPM2.5 on HBSMC migration. **c** Effects of WSPM2.5 on HBSMC migration. **d** Quantitative analysis of HBSMC migration. *, *P* < 0.05 compared with the control

**Fig. 3:**
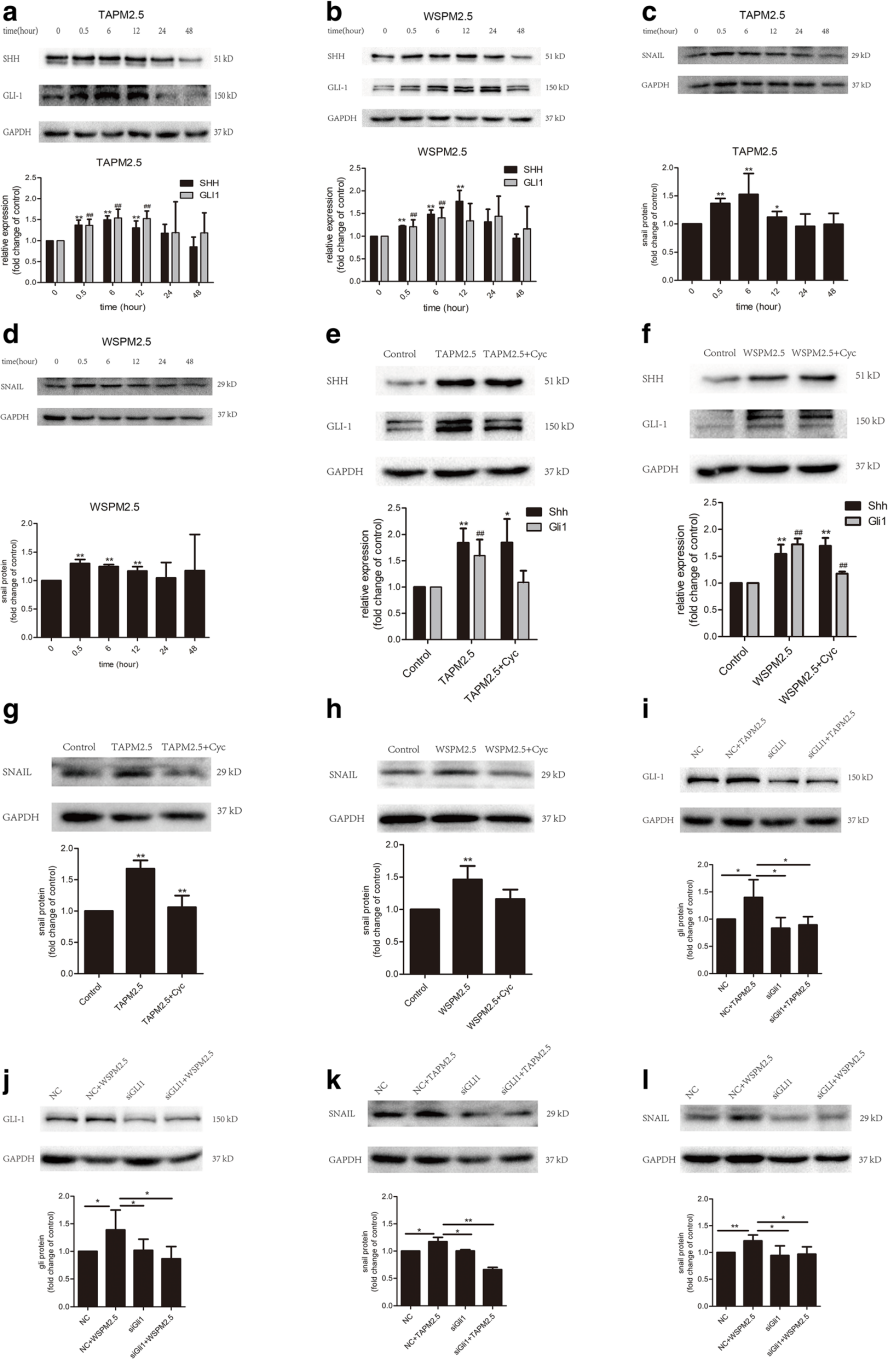
Effects of PM2.5 on the Shh pathway in HBSMCs. **a** TAPM2.5 and (**b**) WSPM2.5 increased Shh and Gli1 expression in HBSMCs in a time-dependent manner. **c** TAPM2.5 and (**d**) WSPM2.5 increased Snail expression in HBSMCs in a time-dependent manner. **e** and **f** Cyclopamine significantly attenuated the effects of TAPM2.5 and WSPM2.5 on Gli1 expression, but had no effect on Shh expression. **g** and **h** Cyclopamine significantly attenuated the effects of TAPM2.5 and WSPM2.5 on Snail expression. **i** and **j** siRNA knockdown of Gli1 significantly attenuated the effects of TAPM2.5 and WSPM2.5 on Gli1 expression. **k** and **l** siRNA knockdown of Gli1 significantly attenuated the effects of TAPM2.5 and WSPM2.5 on Snail expression. *, *P* < 0.05; **, *P* < 0.001; #, *P* < 0.05; ##, *P* < 0.001 compared with the control group

We next characterized the effects of PM2.5 on the migration of HBSMCs and HBFs. As shown in Fig. 2a–d, a 6.6-fold and 9.6-fold increase in HBSMC migration was observed in response to 15 μg/ml TAPM2.5 (Fig. 2b) and 3 μg/ml WSPM2.5 (Fig. 2c), respectively. However, PM2.5 had no significant effect on the migration of HBFs (Additional file 1: Figure S1A–D, Fig. 2e–h). These results provide the first evidence that PM2.5 promotes HBSMC migration.

### Effects of PM2.5 on the Shh pathway in HBSMCs

To elucidate the potential mechanisms by which PM2.5 augments HBSMC migration, we examined the Shh pathway following PM2.5 stimulation. We first checked whether PM2.5 exposure activates the Shh pathway. Interestingly, we observed a dramatic, time-dependent increase in Shh protein expression in TAPM2.5-treated HBSMCs (Fig. 3a). Shh expression increased significantly from 0.5–12 h, and then decreased from 24 to 48 h, following HBSMC treatment with TAPM2.5. Similar changes in Shh expression were observed in WSPM2.5-treated HBSMCs (Fig. 3b). Furthermore, we found that PM2.5-induced Shh activation was accompanied by a significant increase in Gli1 (Fig. 3a and b) and Snail (Fig. 3c and d) protein expression. We further investigated the possibility that Shh directly upregulates Gli1 expression following PM2.5 exposure. The SMO antagonist, cyclopamine, binds to the SMO heptahelical bundle and inhibits its activity, thereby suppressing the Shh pathway. Cyclopamine significantly attenuated Gli1 (Fig. 3e and f) and Snail (Fig. 3g and h) expression. Knocking down Gli1 with siRNA decreased Snail expression (Fig. 3k and l). Taken together, these results suggest that the Shh pathway plays a key role in the response to PM2.5 exposure.

### Effects of the Shh pathway on HBSMC migration

**Fig. 4:**
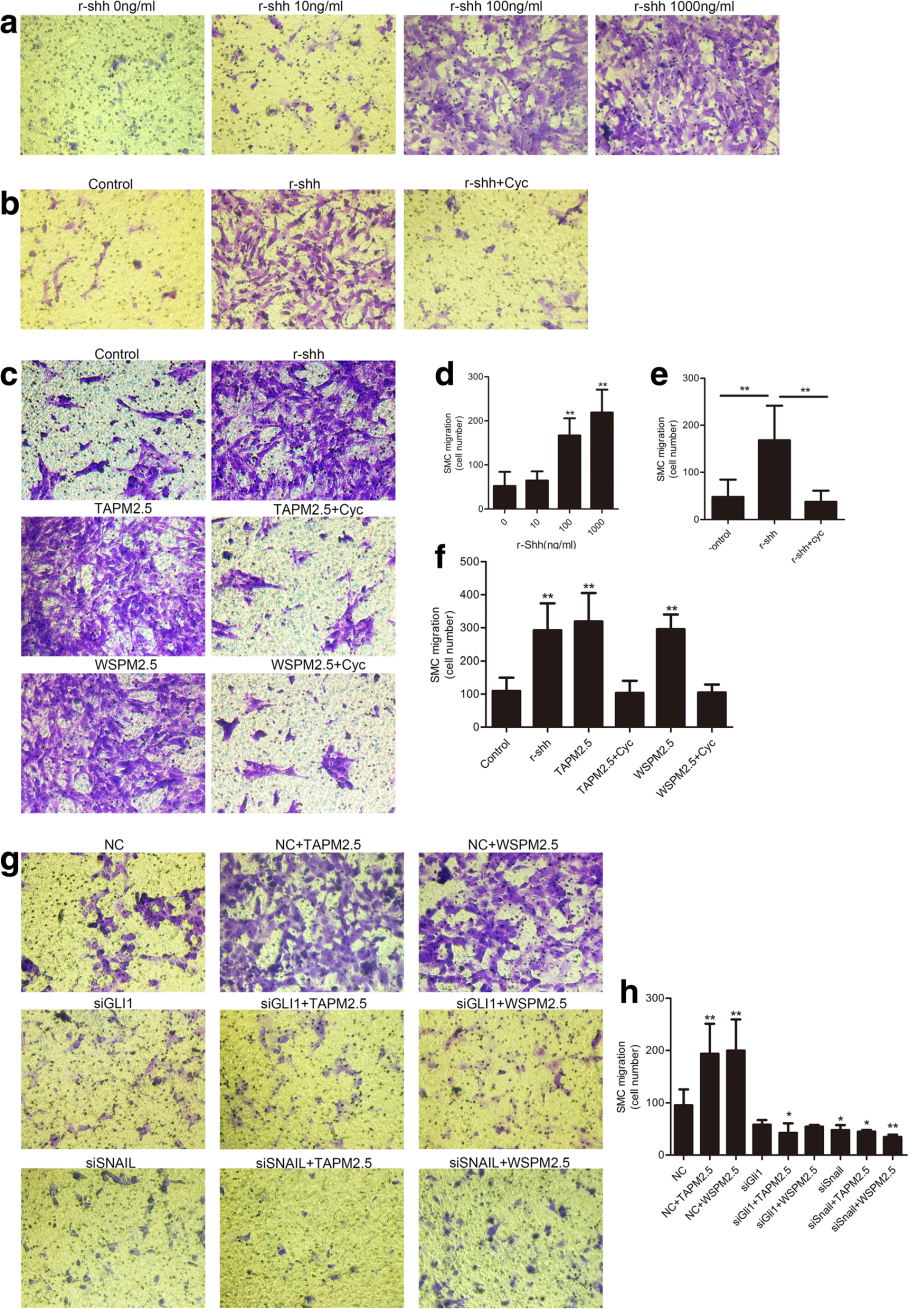
Effects of the Shh pathway on HBSMC migration. **a** and **d** HBSMC migration in response to increasing concentrations of Shh. **b** and **e** Cyclopamine significantly inhibited r-Shh-induced HBSMC migration. **c** and **f** Cyclopamine significantly inhibited TAPM2.5- and WSPM2.5-induced HBSMC migration. **g** and **h** siRNA knockdown of Gli1 or Snail significantly inhibited TAPM2.5- and WSPM2.5-induced HBSMC migration. *, *P* < 0.05; **, *P* < 0.001 compared with the control group

To examine the role of the Shh pathway in the PM2.5-dependent migration of HBSMCs, we performed experiments using r-Shh protein in combination with cyclopamine, or Gli1 and Snail siRNAs. Treatment with r-Shh enhanced HBSMC migration in a dose-dependent manner (Fig. 4a), while cyclopamine significantly inhibited this response (Fig. 4b). Moreover, pretreatment of HBSMCs with cyclopamine (40 μM) attenuated the PM2.5-induced migration (Fig. 4c). Knocking down Gli1 or Snail significantly inhibited the PM2.5-dependent HBSMC migration (Fig. 4g). Taken together, these data suggest that the Shh pathway is necessary for PM2.5-induced HBSMC migration.

## Discussion

Numerous studies have shown that exposure to environmental PM is associated with adverse health effects, including the development of pulmonary disease, cardiovascular disease and cancer, and leads to increased morbidity and mortality. Moreover, our previous studies have demonstrated that exposure to elevated PM concentrations is strongly associated with increased COPD prevalence and reduced respiratory function [9]. Recently, a study by our group has demonstrated that PM2.5 exposure leads to the thickening of the smooth muscle layer in a rat model [21]. To examine the effect of PM2.5 on airway remodeling and to elucidate the underlying mechanisms, we assessed the effect of PM2.5 on several aspects of HBSMC behavior, namely, proliferation, apoptosis and migration.

In this study, we investigated the pro-apoptotic and proliferative effects of PM2.5 on HBSMCs. Our results indicated that a low dose of TAPM2.5 (15 μg/ml) or WSPM2.5 (3 μg/ml) did not induce significant proliferation and that only high concentrations of PM2.5 induced HBSMC apoptosis in vitro. Conversely, a marked increase in cell migration occurred following PM2.5 exposure, and at doses far lower than those required to induce apoptosis. These results demonstrated that HBSMCs were more sensitive to the migratory effects of PM2.5 at low concentrations. According to Gualtieri et al. and Mercer et al., who estimated the bronchial surface area and the deposition of PM2.5 [22, 23], a dose of 15 μg/ml of TAPM2.5 is equivalent to approximately 15 days of atmospheric exposure (assuming a daily exposure of 6 h), and a dose of 3 μg/ml of WSPM2.5 is equivalent to 22 min of rural kitchen wood smoke exposure. Thus, the concentrations that caused HBSMC migration in our experiments can be achieved in practice. The dose of WSPM2.5 required for promoting HBSMC migration was five times lower than that of TAPM2.5, indicating that the migration-inducing potential of WSPM2.5 is greater than that of TAPM2.5. The PM2.5 doses used in our study were non-lethal doses, designed to investigate the effect of PM2.5 on the biological function of normal ASMCs. We showed that cell proliferation and apoptosis did not occur in ASMCs at the low PM2.5 doses, but cell migration occurred significantly. Thus, low doses of PM2.5 stimulated airway remodeling by promoting ASMC migration. Cells that undergo migration change from muscle type to secretory type also have the ability to proliferate and further promote airway remodeling. However, longer time and/or higher PM2.5 doses may be needed, hence further experiments are required. The results of our study are inconsistent with the increase in PM2.5 doses and the severity of COPD clinical manifestations, but only with different emphases. In addition, we believe that the pathogenesis of COPD is caused by low-dose and long-term exposure to PM2.5.

In our study low doses of PM2.5 promoted HBSMC migration, but failed to stimulate HBF migration, suggesting that PM2.5 plays a major role in the migration of HBSMCs at low doses, which is a unique phenomenon in the airway structure. However, HBFs are important airway structural cells, whose proliferation and secretion function contribute to airway fibrosis leading to airway remodeling. HBFs also have the ability to migrate. It has been reported that HBFs differentiate into HBSMCs under some external stimulation, thereby promoting muscle layer thickening. However, under our exposed experimental conditions, HBFs were less sensitive than HBSMCs. The effect of PM2.5 as an important pathogenic factor on HBFs needs to be further studied, and our group has already carried out some related studies.

Our experimental results can be explained by the effects of Snail genes, which have been linked to cell migration. Analysis of Snail expression and function in triploblasts (animals with three germ layers) of the Lophotrochozoan, Ecdysozoan and Deuterostome families supports a role for Snail in regulating cell migration. For instance, it has been shown that Snail homologues were not expressed in the mesoderm, but rather in ectodermal tissues that undergo changes in cell shape or morphogenetic movements [24, 25]. Similarly, Snail is expressed in the developing skin of mice when skin cells invaginate to form hair follicle buds [26]. In conclusion, these data suggest that Snail can regulate cell migration. Although they play a crucial role in embryonic development, Snail genes can have pathological effects in the adult. In embryos, the Snail-mediated induction of cell migration facilitates the generation of different tissues and organs that are located far from the site of origin of their precursors [17]. In cancer, however, Snail genes facilitate the delamination of cells from the primary tumor and to metastasize in other parts of the body. Snail is upregulated in non-small cell lung cancer (NSCLC), which is associated with poor prognosis, and promotes tumor progression in vivo. Moreover, it has been reported that overexpression of Snail leads to upregulation of secreted proteins, acidic and rich in cysteine (SPARC) in models of premalignancy and established disease, as well as in lung carcinoma tissues in situ [27]. Snail expression was generally high in all subjects throughout the airway wall with marked cytoplasmic to nuclear shift in COPD. Furthermore, Snail expression was associated with airflow obstruction and with expression of a canonical EMT biomarker (S100A4) [28]. The Snail pathway plays critical roles in pulmonary fibrosis animal models [29, 30]. In COPD, Snail genes facilitate ASMC migration into the airway intima, where they can secrete a series of factors leading to airway remodeling.

We next investigated the molecular mechanism by which PM2.5 promotes HBSMC migration. We examined the effects of PM2.5 on the expression of proteins associated with the Shh pathway. Obstructive airway diseases such as COPD and asthma are also associated with altered Hh signaling [10]. Growing evidence has highlighted the importance of the Shh pathway in cell migration [31–33]. The canonical Shh pathway, which depends on SMO and the transcription factor Gli, is also implicated in cell migration via gene regulation. In addition, when Gli1 expression is induced in epithelial cells, Snail is rapidly and dramatically upregulated [34]. In pancreatic cancer cells, knocking out Gli1 reduces Snail expression [35]. Inhibiting the Hh pathway with cyclopamine, a steroid that blocks SMO, decreases Gli1 and Gli2 expression, as well as Snail expression [36]. The Shh pathway has also been found to be reactivated and associated with cell migration in lung cancer [37, 38]. In summary, previous studies have revealed the involvement of the Shh pathway in cancer cell migration. However, little is known about the role of the Shh pathway in HBSMC migration. Thus, there is an urgent need to identify the mechanisms of HBSMC migration.

Our data show that PM2.5 caused a dramatic, time-dependent increase in Shh protein expression. Similar effects were observed for Gli1 and Snail expression. Intriguingly, Shh pathway activity increased significantly from 0.5–12 h, and then decreased from 24 to 48 h. The reason for this pattern remains unclear, but it is possible that Shh signaling activates other pathways, resulting in a complex network of signaling events following PM2.5 exposure. These interesting possibilities require further investigation. Our results demonstrated the importance of Shh in the response to PM2.5 exposure, given that inhibition of Shh signaling by cyclopamine was able to downregulate Gli1 and Snail expression, thereby inhibiting the effects of PM2.5 on HBSMC migration. Taken together, these results indicate that PM2.5 exposure activates the Shh signaling pathway.

**Fig. 5:**
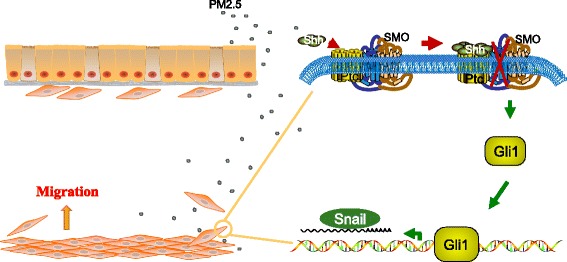
Schematic depicting the migration signaling pathway induced by PM2.5 in HBSMCs. Here, we showed that PM2.5 regulated HBSMC migration by activating the Shh signaling pathway. PM2.5 increases the expression of Shh. Shh binds to Ptch and inhibits the repression of SMO, thereby activating Gli1. Gli1 translocates to the nucleus and activates Snail transcription, triggering HBSMC migration to the epithelium

Furthermore, our results demonstrated that PM2.5-induced migration was accompanied by activation of the Shh pathway. r-Shh induced HBSMC migration in a dose-dependent manner, and cyclopamine significantly reduced this effect. Moreover, cyclopamine attenuated the PM2.5-induced HBSMC migration. Knocking down Gli1 or Snail expression also significantly inhibited HBSMC migration (Fig. 4). Based on existing evidence and our current data, we propose that the Shh pathway plays a vital role in PM2.5-induced HBSMC migration (Fig. 5).

## Conclusion

To the best of our knowledge, this is the first study to establish that the Shh signaling pathway is a promising therapeutic target for preventing PM2.5-induced HBSMC migration. Further studies and a greater understanding of the changes that occur in the airway are required to elucidate how airway remodeling and ASMC migration are involved in the pathology of COPD, and whether early therapeutic interventions will prevent the progression of airway remodeling caused by PM2.5 exposure.

## Additional files

Additional file 1: **Figure S1A–D.** Effects of PM2.5 on the migration of HBFs. (A) HBF migration in the control group. (B) Effects of TAPM2.5 on HBF migration. (C) Effects of WSPM2.5 on HBF migration. (D) Quantitative analysis of HBF migration. *, *P* < 0.05 compared with the control. (TIFF 9170 kb)

## Abbreviations

ASMCs: Airway smooth muscle cells
COPD: Chronic obstructive pulmonary disease
HBFs: Human bronchial fibroblasts
HBSMC: Human bronchial smooth muscle cell
PM2.5: Fine particulate matter
r-Shh: Recombinant Shh protein
Shh: Sonic hedgehog
TAPM2.5: Traffic ambient PM2.5
WSPM2.5: Wood smoke PM2.5
