# Supplementary figures and images for: PM2.5 promotes human bronchial smooth muscle cell migration via the sonic hedgehog signaling pathway
Source: Respir Res. 2018 Mar 2;19:37. doi: 10.1186/s12931-017-0702-y (PMC5833105; doi:10.1186/s12931-017-0702-y)

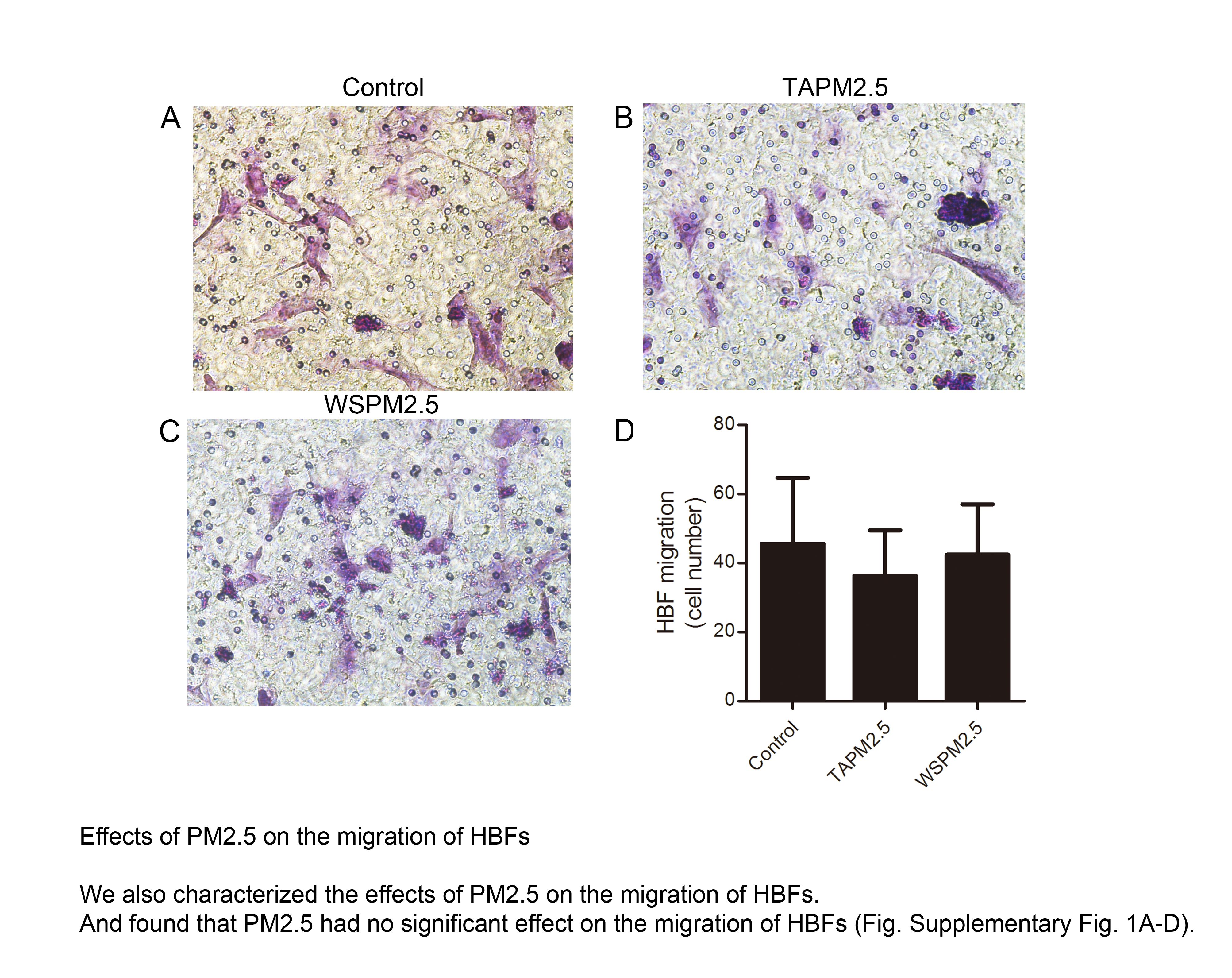

Supplement: Supplementary file 1 — Figure S1A–D. Effects of PM2.5 on the migration of HBFs. (A) HBF migration in the control group. (B) Effects of TAPM2.5 on HBF migration. (C) Effects of WSPM2.5 on HBF migration. (D) Quantitative analysis of HBF migration. *, P < 0.05 compared with the control. (TIFF 9170 kb) [file 12931_2017_702_MOESM1_ESM.tif]
